# Prediction and Analysis of Key Genes in Glioblastoma Based on Bioinformatics

**DOI:** 10.1155/2017/7653101

**Published:** 2017-01-16

**Authors:** Hao Long, Chaofeng Liang, Xi'an Zhang, Luxiong Fang, Gang Wang, Songtao Qi, Haizhong Huo, Ye Song

**Affiliations:** ^1^Department of Neurosurgery, Nanfang Hospital, Southern Medical University, Guangzhou, Guangdong 510515, China; ^2^Department of Neurosurgery, The Third Affiliated Hospital of Sun Yat-sen University, Guangzhou 510665, China; ^3^Department of General Surgery, Shanghai Ninth People's Hospital Affiliated to Shanghai Jiao Tong University School of Medicine, Shanghai 200011, China

## Abstract

Understanding the mechanisms of glioblastoma at the molecular and structural level is not only interesting for basic science but also valuable for biotechnological application, such as the clinical treatment. In the present study, bioinformatics analysis was performed to reveal and identify the key genes of glioblastoma multiforme (GBM). The results obtained in the present study signified the importance of some genes, such as COL3A1, FN1, and MMP9, for glioblastoma. Based on the selected genes, a prediction model was built, which achieved 94.4% prediction accuracy. These findings might provide more insights into the genetic basis of glioblastoma.

## 1. Introduction

Glioblastomas are highly invasive tumors associated with high levels of mortality in the central nervous system, and their symptoms include bloating, pelvic pain, difficult eating, and frequent urination. It is difficult to diagnose glioblastoma at its early stages (I/II) as most symptoms of this disease are nonspecific [1]. Glioblastoma is a rare disease, with a rate of 2-3 cases per 100,000 person life-years in Europe and North America [2], accounting for 77–80% of primary malignant tumors of the brain. Among the patients diagnosed with glioblastoma, approximately 50% die within one year, while 90% die within three years [3]. Due to the great threat of glioblastoma to human health, the treatment of glioblastoma remains a major challenge.

Over the past years, tremendous genomics and proteomics studies have been conducted to explore the molecular mechanisms underlying the development and progression of glioblastoma. The characterization of glioblastoma has provided invaluable data related to this molecularly heterogeneous disease. Recent advances in high-throughput microarrays have received extensive attention and made substantial progress in reconstructing the gene regulatory network of medical biology [4–11]. Using microarray analysis, significant differences in gene expression between normal and disease tissues have been observed. However, as a result of the underlying shortcomings of microarray technology, such as small sample size, measurement error, and information insufficiency, unveiling this disease mechanism has remained a major challenge to glioblastoma research. Hence, GO, pathway information, network-based approaches, and machine learning algorithms have been employed to identify the mechanisms underlying this disease.

In the present study, we identified the differentially expressed genes (DEGs) between the glioblastoma samples and normal brain samples. In addition, eleven significant target genes for diagnosing glioblastoma were identified based on GO processes, KEGG pathways, and protein-protein interaction networks. Based on the results, a prediction model was built with a prediction accuracy of 94.4% with these eleven genes using Bayes net.

## 2. Materials and Methods

### 2.1. Data Preparation

**Figure 1:**
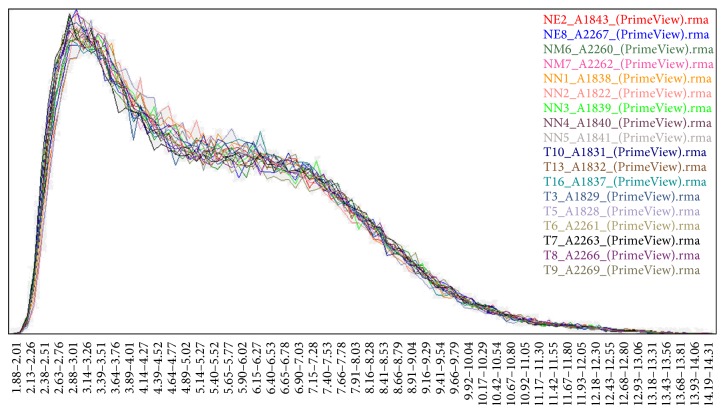
Histogram of the raw fluorescence intensity data.

The datasets available in this analysis contained 18 samples, including 9 glioblastoma tissue samples and 9 normal brain tissue samples from epilepsy surgery. All specimens had confirmed pathological diagnosis and were classified according to the World Health Organization (WHO) criteria. All the tumor samples were obtained from primary surgery. For the use of these clinical materials for research purposes, prior consent from patients and approval from the Ethics Committees of Nanfang Hospital (number 2013105) were obtained. These data (CEL form) and annotation files were collected for further analysis.  Figure 1 shows that the gene expression signals for the 18 samples fit well with each other and could be employed in the bioinformatics analysis in the present study.

## 3. Results

### 3.1. Raw Data

Limma package in R was used to identify the DEGs between the glioblastoma samples and the normal controls. According to the cut-off criteria of |log FC| > 2.0 and *p* value < 0.05, we obtained 2365 DEGs, including 1021 up- and 1344 downregulated genes (please visit the following website for more raw data information: https://www.ncbi.nlm.nih.gov/geo/query/acc.cgi?acc=GSE90886).

### 3.2. Gene Ontology Analysis

**Figure 2:**
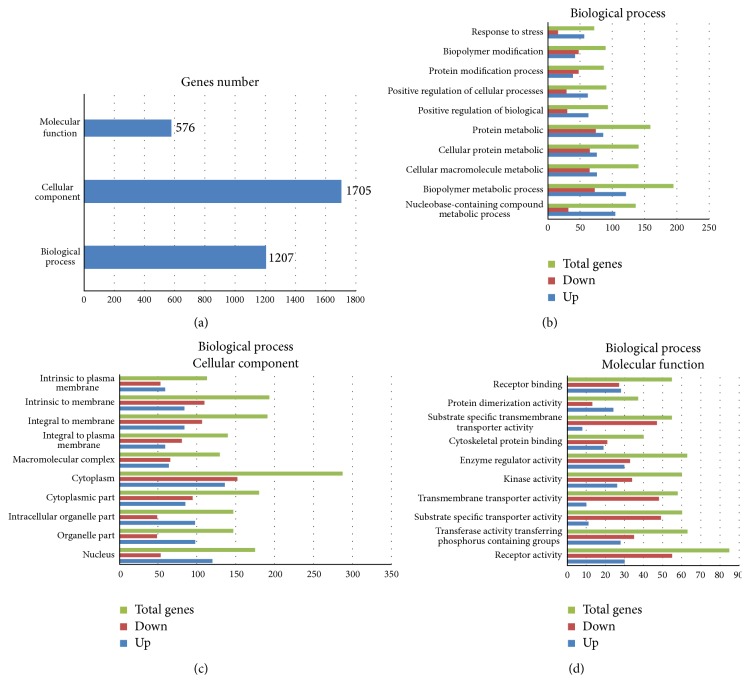
(a) GO enrichment of DEGs. (b) DEGs in BP. (c) DEGs in CC. (d) DEGs in MF.

GO analyses were performed by DAVID which demonstrated that the majority of DEGs were enriched in cellular components, cytoplasm, integral to membrane, intrinsic to membrane, biopolymer metabolic processes, cytoplasmic parts, and nucleus (Figure 2). The upregulated genes were significantly enriched in cytoplasm, nucleus, nucleobase-containing compounds, metabolic processes, and biopolymer metabolic processes.

### 3.3. Analysis of KEGG Pathways

To obtain further insight into the functions of DEGs, DAVID was applied to identify the significant dysregulated KEGG pathways. The pathways obtained with a *p* value < 0.05 and a gene count > 2 for the up- and downregulated genes were collected (Table 1). According to the enrichment results, the genes were significantly enriched in following pathways: cancer pathways, regulation of the actin cytoskeleton, the MAPK signaling pathway, focal adhesion, the calcium signaling pathway, ECM-receptor interaction, long-term potentiation, endocytosis, leukocyte transendothelial migration, and the p53 signaling pathway. Among these pathways, the upregulated genes were significantly enriched in the pathways of focal adhesion, cancer, ECM-receptor interaction, MAPK signaling, and p53 signaling. The downregulated DEGs were enriched in the pathways of calcium signaling, MAPK signaling, endocytosis, regulation of actin cytoskeleton, and long-term potentiation.

### 3.4. PPI Network Construction

**Figure 3:**
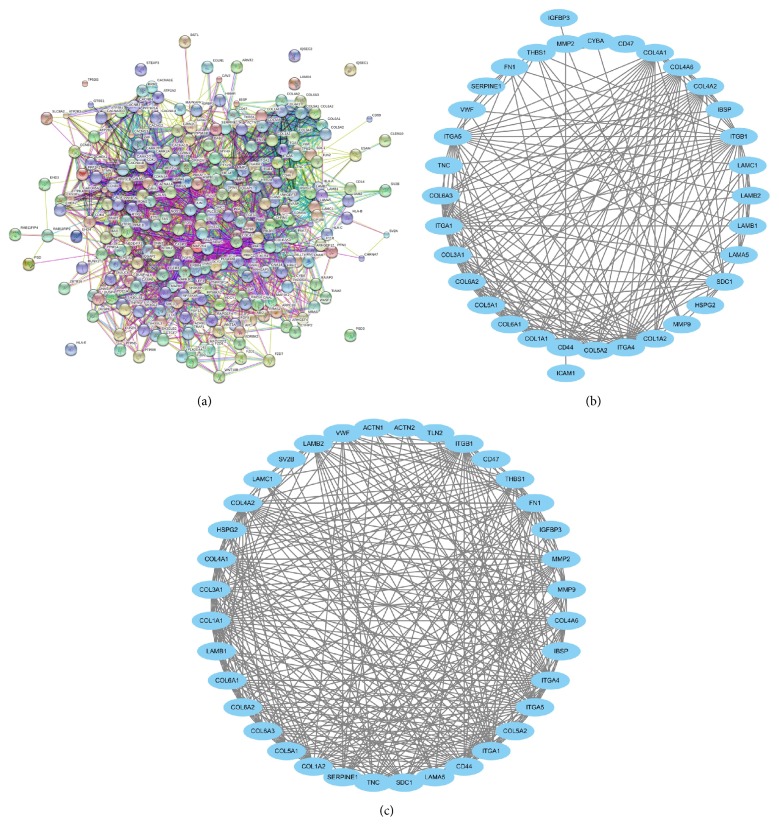
(a) Protein-protein interaction networks of the corresponding DEGs. ((b) and (c)) Modules of the PPI network.

The STRING tool was used to determine the PPI relationships of the DEGs. In total, 2182 PPI relationships were obtained with a combined score >0.4. After filtering out the nodes of degree ≤5, we constructed a network with 240 nodes and 2182 edges (Figure 3(a)).

Based on the PPI network constructed above, PPI network enrichments were performed. The results revealed 5 enriched modules with a size >5 and a* p* < 0.05. Among the five modules, two significant enrichments, Module A and Module B, are shown in Figures 3(b) and 3(c). According to Figure 3(b), it is difficult to determine which module is better, as they had similar sizes and edges. However, as Module A has 38 nodes and 340 edges compared with Module B with 36 nodes and 320 edges, we considered Module A as the better module.

To investigate the biological functions of the genes in Module A, GO functional enrichments were performed using STRING tools. A total of 31 genes in Module A were significantly enriched in biological processes and cellular components, such as extracellular matrix organization, extracellular structure organization, extracellular region part, locomotion, and cell movement or subcellular components. Subsequently, these 31 genes were further investigated using KEGG pathway enrichment analysis. The results showed that the genes in Module A were primarily enriched by the following pathways: ECM-receptor interaction, focal adhesion, the PI3K-Akt signaling pathway, amoebiasis, protein digestion/absorption, and pathways in cancer.

The connectivity degree of each node of the PPI network was calculated, and the results of some nodes are shown in Table 2. As shown in Table 2, several genes, including MMP9, CD44, COL1A1, COL1A2, CAMK2A, and CAMK2B, exhibited a high connectivity degree >25. Hence, these genes were selected as key nodes and might play important roles in the progression of GBM.

### 3.5. Prediction Model

Based on the selected eleven genes, a predictive glioblastoma model was constructed using Bayes net algorithm. To validate the predictive capability of the model, a leave-one-out (LOO) cross-validation test, widely used in prediction-related problems, was adopted in the present study. For the LOO cross-validation test tests, the datasets were randomly divided into 18 subsets. Each classifier was constructed using the samples from seventeen of the subsets and the samples in the remaining subset were treated as untrained data, which were used in the prediction as independent test samples. Each subset was omitted when constructing the classifier and predicted in turn. The total prediction accuracy was obtained after averaging the correct prediction rates of the 18 data subsets. The following prediction results were obtained using the Bayes net method: SN: 88.9%, SP: 100%, ACC: 94.4%, and MCC: 0.795.

## 4. Discussion

In the present study, we obtained 2365 genes, including 1021 upregulated genes and 1344 downregulated genes using gene expression profiling. Among the 2365 genes identified, there were 365 differentially expressed genes, including 237 upregulated genes and 124 downregulated genes. Most of these genes were enriched in ten pathways, including MAPK signaling, cancer, focal adhesion, calcium signaling, actin cytoskeleton regulation, endocytosis, ECM-receptor interaction, leukocyte transendothelial migration, long-term potentiation, and p53 signaling pathways. Moreover, the upregulated DEGs were primarily enriched in pathways in cancer, focal adhesion, and ECM-receptor interaction, while the downregulated DEGs were significantly related to pathways, such as the calcium signaling pathway, MAPK signaling pathway, and endocytosis. COL3A1, MMP9, CAMK2A, CD44, HTR2A, SV2B, GRIN2A, COL6A3, and SH3GL3 have been identified as significant genes in these pathways. MMP9, FN1, FGF13, and COL4A2 are significant genes in the pathways associated with cancer. COL3A1, COL6A3, COL1A2, FN1, and TNC are significant genes in the focal adhesion pathway. CAMK2A, HTR2A, and GRIN2A are significant genes in the calcium signaling pathway. COL3A1, CD44, SV2B, and COL6A3 are significant genes in ECM-receptor interactions.

These results indicate that the ECM-receptor interaction pathway is a significant pathway enriched by upregulated DEGs. In the present study, COL3A1 and CD44 in ECM-receptor interaction pathway were significantly upregulated. CD44, an unclassified cell adhesion molecule, is involved in cell-cell interactions, cell adhesion, and migration [12, 13]. Studies have shown that CD44 participates in a wide variety of cellular functions, including lymphocyte activation and the recirculation, recurrence, and development of tumors [14]. In a previous study, Yoshida indicated that the overexpression of CD44 was important for the growth and survival of glioblastomas, and the monoclonal anti-CD44 antibody affects the migration of glioblastoma cells [15, 16]. COL3A1 encodes fibrillar collagen, a major component of the extracellular matrix protein surrounding cancer cells [17, 18]. The presence of ECM protein prevents the apoptosis of cancer cells. COL3A1 plays an important role in apoptosis, proliferation regulation, and anticancer drug resistance [19], indicating that the ECM-receptor interaction pathway plays an important role in GBM, and CD44 and COL3A1 might be potential diagnostic and therapeutic targets in this disease.

In the present study, MMP9 and FN1, key proteins in cancer pathways, were also upregulated. The proteins of the matrix metalloproteinase (MMP) family are involved in the breakdown of extracellular matrix in normal biological processes, such as embryonic development, angiogenesis, cell migration, intracerebral hemorrhage, and metastasis [20, 21]. As a member of the MMPs, MMP9 is involved in the degradation of the extracellular matrix. MMP9 also plays roles in tumor development, as these proteins facilitate extracellular matrix remodeling and participate in angiogenesis. Forsyth et al. reported the involvement of MMP9 in different aspects of the pathophysiology of malignant gliomas by remodeling associated with neovascularization [22]. Choe et al. detected MMP9 in the tumor samples of GBM patients but not in normal brain tissue samples. Moreover, these authors also showed that EGFRvIII overexpression affects MMP9 activation by the activation of MAPK/ERK [23]. FN1, a high-molecular weight glycoprotein of the extracellular matrix, binds extracellular matrix components, such as collagen, fibrin, and heparan sulfate proteoglycans. Wang et al. reported that FN is involved in the maintenance of integrin b1 fibronectin receptors in glioma cells and could be regarded as an important mediator [24]. Han et al. proposed that fibronectin stimulates non-small cell lung carcinoma cell growth and survival through the activation of the Akt/mTOR/p70S6K pathway [25], and recently, fibronectin has been implicated in carcinoma development as a potential biomarker for radioresistance [14].

Yu and Stamenkovic identified a functional relationship between the hyaluronan receptor CD44, MMP9, and transforming growth factor-beta in the control of tumor-associated tissue remodeling [26, 27]. These authors also showed that several isoforms of CD44, expressed on murine mammary carcinoma cells, provide cell surface docking receptors for proteolytically active MMP9. The localization of MMP9 on the cell surface is required to promote tumor invasion and angiogenesis. Moreover, the cell surface expression of MMP9 stimulated the formation of capillary tubes by bovine microvascular endothelial cells.

## 5. Conclusions

The results of the present study suggested that glioblastoma is closely associated with the dysregulation of the pathways in cancer, MAPK signaling, focal adhesion, and calcium signaling. In addition, we also identified key genes, including MMP9, CD44, CDC42, COL1A1, COL1A2, CAMK2A, and CAMK2B, as potential target genes for diagnosing glioblastoma.

## Figures and Tables

**Table 1:** DEG pathway distribution.

KEGG pathway	DEGs	Upregulation	Downregulation
Calcium signaling pathway	45	4	41
MAPK signaling pathway	61	24	37
Endocytosis	37	11	26
Regulation of actin cytoskeleton	44	19	25
Long-term potentiation	24	2	22
Pathways in cancer	57	41	16
Focal adhesion	48	36	12
Leukocyte transendothelial migration	29	19	10
ECM-receptor interaction	33	29	4
p53 signaling pathway	22	22	0

**Table 2:** The statistical results of the connectivity degrees of the PPI network.

Gene	Degree	Differential rates
CDC42	73	−6.928589
MMP9	49	16.665218
CD44	41	14.821273
CAV1	39	5.418803
THBS1	35	6.9339356
CAMK2B	33	−7.2954154
CAMK2A	32	−14.86015
COL1A2	30	9.719963
FN1	28	9.536128
COL4A2	27	7.803446
COL3A1	26	27.03572
